# EEG and fMRI evidence for autobiographical memory reactivation in empathy

**DOI:** 10.1002/hbm.25557

**Published:** 2021-06-14

**Authors:** Federica Meconi, Juan Linde‐Domingo, Catarina S. Ferreira, Sebastian Michelmann, Bernhard Staresina, Ian A. Apperly, Simon Hanslmayr

**Affiliations:** ^1^ School of Psychology University of Birmingham Birmingham; ^2^ Max Plank Institute Berlin for Human Development Berlin Germany; ^3^ Princeton Neuroscience Institute Princeton New Jersey USA; ^4^ Center for Human Brain Health University of Birmingham Birmingham UK; ^5^ Institute for Neuroscience and Psychology University of Glasgow Glasgow Scotland

**Keywords:** autobiographical memory, EEG, EEG pattern classifier, empathy, fMRI

## Abstract

Empathy relies on the ability to mirror and to explicitly infer others' inner states. Theoretical accounts suggest that memories play a role in empathy, but direct evidence of reactivation of autobiographical memories (AM) in empathy is yet to be shown. We addressed this question in two experiments. In Experiment 1, electrophysiological activity (EEG) was recorded from 28 participants. Participants performed an empathy task in which targets for empathy were depicted in contexts for which participants either did or did not have an AM, followed by a task that explicitly required memory retrieval of the AM and non‐AM contexts. The retrieval task was implemented to extract the neural fingerprints of AM and non‐AM contexts, which were then used to probe data from the empathy task. An EEG pattern classifier was trained and tested across tasks and showed evidence for AM reactivation when participants were preparing their judgement in the empathy task. Participants self‐reported higher empathy for people depicted in situations they had experienced themselves as compared to situations they had not experienced. A second independent fMRI experiment replicated this behavioural finding and showed increased activation for AM compared to non‐AM in the brain networks underlying empathy: precuneus, posterior parietal cortex, superior and inferior parietal lobule, and superior frontal gyrus. Together, our study reports behavioural, electrophysiological, and fMRI evidence that robustly supports AM reactivation in empathy.

## Graphical abstract

Theoretical accounts suggest that memories play a role in empathy, but direct evidence of reactivation of autobiographical memories (AM) in empathy is yet to be shown. We addressed this question in one EEG and one fMRI experiment. Participants self‐reported higher empathy for people depicted in situations they had experienced themselves as compared to situations they had not experienced. An EEG pattern classifier showed evidence for AM reactivation when participants were preparing their judgement in the empathy task. Increased activation was observed for AM compared to non‐AM in the brain networks underlying empathy. Together, our study reports behavioural, electrophysiological, and fMRI evidence that robustly supports AM reactivation in empathy.

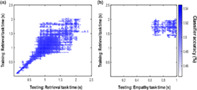

**Abbreviations**
AM: autobiographical memory
IPL: inferior parietal lobule
LDA: linear discriminant analysis
non‐AM: non‐AM
PCC: posterior cingulate cortex
PHG: parahippocampal gyrus
SFG: superior frontal gyrus
SPL: superior parietal lobule
TPJ: temporoparietal junction

## 1 INTRODUCTION

When we encounter somebody, who has a physical injury, like a broken leg, we feel we have a good understanding of their pain, especially if we have experienced that same injury in our life. Therefore, it is intuitively compelling to assume that empathy, that is, the ability to share and understand others' inner states, draws on first‐hand experiences we collected in our past, that is, autobiographical memories (AM). However, this compelling intuition cannot be taken for granted because empathy might instead be supported by semantic memory about the general experience of pain and the conditions in which it is likely to occur (Perry, Hendler, & Shamay‐Tsoory, 2011; Rabin & Rosenbaum, 2012). The present study used advanced imaging methods to distinguish carefully between the roles of autobiographical and semantic memory, and seek the first direct evidence of reactivation of AM in the service of empathy.

Empathy is a rich and multifaceted process. On the one hand, empathy can occur through a mechanism of inner resonance of others' inner states, that is, sharing and simulating others' experience. On the other hand, it can entail the explicit understanding and inference of the others' minds and thoughts, that is, mentalizing. These components of empathy have also been referred to as “affective empathy,” or “hot empathy” and “cognitive empathy,” or “cold reasoning,” respectively (Amodio & Frith, 2006; Davis & Kraus, 1991; Krämer, Mohammadi, Doñamayor, Samii, & Münte, 2010). They have different time‐courses with the first modulating event‐related potentials (ERPs) within 250 ms and the second modulating mainly the P300 (Fan & Han, 2008; Meconi, Doro, Lomoriello, Mastrella, & Sessa, 2018; Palmieri et al., 2021; Sessa, Meconi, Castelli, & Dell'Acqua, 2014; Sessa, Meconi, & Han, 2014). Neuroimaging and lesion studies have shown anatomical and functional dissociation between affective and cognitive empathy. Somatosensory areas and the human mirror neuron system subserve affective empathy; it helps us feel the other's emotion as if we were feeling it first‐hand. Medial and dorsolateral prefrontal cortices, temporal poles, and parietal areas, such as precuneus, posterior cingulate, and temporoparietal junction (TPJ), subserve cognitive empathy. Cognitive empathy helps us build an explicit representation of the other's mind via imagination, perspective taking, or reasoning about others (Frith & Frith, 2003; Lamm, Rütgen, & Wagner, 2019; Rütgen et al., 2015, 2020; Samson, Apperly, Chiavarino, & Humphreys, 2004; Shamay‐Tsoory, Aharon‐Peretz, & Perry, 2009; Zaki & Ochsner, 2012; Zaki, Wager, Singer, Keysers, & Gazzola, 2016). Recent meta‐analyses have confirmed these anatomical dissociations (Lamm, Decety, & Singer, 2011; Molenberghs, Cunnington, & Mattingley, 2012; Molenberghs, Johnson, Henry, & Mattingley, 2016).

The claim for an interplay between AM and empathy is supported by several sources of convergent evidence. Ciaramelli, Bernardi, and Moscovitch (2013) showed that healthy participants use memory for fictional others' past experiences to imagine how they might feel as they face similar situations. Healthy students show higher empathy levels for adults experiencing chronic pain if they can rely on their own general AM of physical pain when compared to control conditions (Bluck, Baron, Ainsworth, Gesselman, & Gold, 2013). Patients with congenital insensitivity to pain show attenuated self‐rated empathy for others' pain, for which they could have collected no experience or memory (Danziger, Faillenot, & Peyron, 2009). Several studies provide evidence of common brain networks for AM retrieval and cognitive empathy (Buckner & Carroll, 2007; Spreng & Grady, 2009; Spreng, Mar, & Kim, 2008). with these brain areas including precuneus, posterior cingulate cortex (PCC), retrosplenial cortex, medial temporal lobe (MTL), TPJ, and medial prefrontal cortex (mPFC, BA 10). Neuropsychological studies on patients with different memory impairments have reported generally convergent results about impoverished empathic abilities. This was measured by neuropsychological assessments or self‐report questionnaires entailing one or both components of empathy. Empathy deficits are co‐morbid symptoms of several psychiatric disorders with long‐term memory impairment, for example, schizophrenia (Corcoran & Frith, 2003; Meconi et al., 2016). They have been directly observed in patients with Alzheimer's disease (Moreau, Viallet, & Champagne‐Lavau, 2013; Ramanan et al., 2017), Korsakoff's syndrome (Drost, Postma, & Oudman, 2019; Oosterman, Derksen, van Wijck, Veldhuijzen, & Kessels, 2011), mild cognitive impairment (Moreau et al., 2015), Parkinson disease (Monetta, Grindrod, & Pell, 2009; Pell et al., 2014; Xi et al., 2015), and semantic dementia (Duval et al., 2012). In healthy ageing affective empathy seems to decrease with age (Chen, Chen, Decety, & Cheng, 2014; Duval, Piolino, Bejanin, Eustache, & Desgranges, 2011; Ze, Thoma, & Suchan, 2014). However, conclusions from the latter studies must be treated with some caution because it is unclear which factor might be responsible for any observed empathy deficit in clinical populations where memory deficits co‐occur with global cognitive decline (see also Hillis, 2014).

Curiously, the handful of studies on patients with amnesia, that is, a memory disorder in which the ability to consciously access AM is impaired by focal hippocampal cortices damage, showed that cognitive, but not affective, empathy is spared (Rosenbaum, Stuss, Levine, & Tulving, 2007) or only mildly impaired (Beadle, Tranel, Cohen, & Duff, 2013; Staniloiu, Borsutzky, Woermann, & Markowitsch, 2013). While studies of amnesia are potentially powerful sources of evidence evaluating the role of memory in empathy, they do not provide definitive evidence about the role of AMs. This is because the retrieval of AMs does not rely only on the hippocampal cortices but is underpinned by a network of brain areas that involves the prefrontal cortex and parietal areas including precuneus, posterior parietal cortex and the retrosplenial cortex (Boccia, Teghil, & Guariglia, 2019; Cabeza & St Jacques, 2007; Cotelli et al., 2012). In line with the systems consolidation account, by which memories become gradually independent of the hippocampus and stably stored in the neocortex, at least remote memories might still be available as a source of semantic knowledge or implicit memories for the patients (Antony, Ferreira, Norman, & Wimber, 2017; McClelland, McNaughton, & O'Reilly, 1995). This clearly leaves open the possibility that AMs are retrieved during cognitive empathy, and that this could be detected with appropriate methods.

Even though the studies mentioned above support the idea that cognitive empathy draws on AM, critical evidence is missing. In particular, it has not yet been demonstrated that AMs are actively retrieved in the service of empathy. To test for evidence of a re‐activation of AM when empathizing with others, we investigated healthy adults' empathy for an AM experience that the participants shared with the targets of empathy, in contrast with an un‐shared experience for which participants had no AM.

**FIGURE 1:**
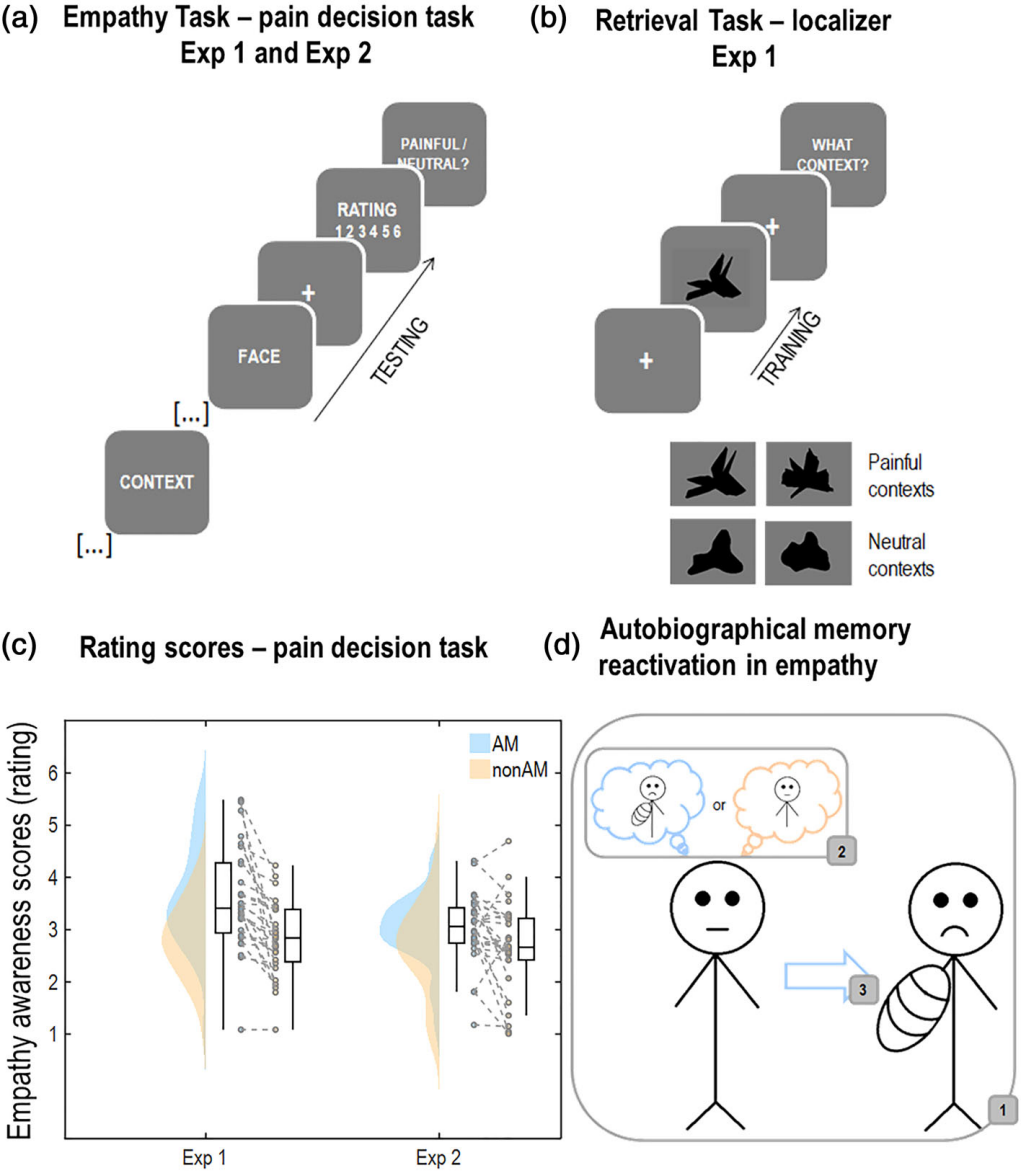
Experimental design. (a) Schematic representation of the empathy task used in Experiments 1 and 2. Participants were required to rate how much empathy they felt for the person depicted in the preceding context. (b) Schematic representation of the retrieval task used in Experiment 1 that was used to train the linear discriminant analysis (LDA) classifier. Participants first learnt to associate four abstract figures with the same sentences describing painful contexts presented during the empathy task (not shown here). In the actual task, for each trial, participants were presented with one of the four figures and had to picture in their mind's eye the context that they learnt to associate with that specific figure. (c) Raincloud plots of the subjective reports of participants' empathy awareness in Experiments 1 and 2. (d) Concept of the study; when we encounter someone, who shares our same physically painful experience, the memory of that experience is reactivated to empathize

Recent advances in multivariate pattern analysis methods show that brain activity patterns can be tracked during the encoding of new neutral episodes and re‐observed during their retrieval (Linde‐Domingo, Treder, Kerrén, & Wimber, 2019; Michelmann, Bowman, & Hanslmayr, 2016). Furthermore, recent neuroimaging studies show that reinstatement of autobiographical pain involves partial reinstatement of activity in the brain areas that process nociception (Fairhurst, Fairhurst, Berna, & Tracey, 2012; Forkmann, Wiech, Sommer, & Bingel, 2015). Therefore, we here tested for a direct evidence of online reactivation of AM when participants were required to explicitly rate their empathy awareness for others' neutral and painful experiences in two independent experiments (Figure 1c,d). In Experiment 1, EEG was recorded during two sequential tasks. The first was a pain decision task, classically used to prompt an empathic reaction, in which targets for empathy were depicted in contexts for which participants either did or did not have an AM (Figure 1a). The second was a task that explicitly required retrieval of the AM and non‐AM contexts. The retrieval task was used to extract the neural fingerprints of AMs and non‐AMs (Figure 1b). A linear discriminant analysis (LDA) EEG pattern classifier was trained during the retrieval task and tested on data obtained from the preceding empathy task to test for the online reactivation of the memories in explicit empathy. In Experiment 2, fMRI was measured from an independent sample performing the same empathy task to test if AM would show increased activation in brain areas robustly associated with the cognitive empathy network when compared to non‐AM replicating and further support the role of AM in the service empathy.

## 2 MATERIALS AND METHODS

The protocol for both experiments was approved by the University of Birmingham Research Ethics Committee (ERN‐16‐0101A). Written informed consent was obtained from all participants for both experiments.

### 2.1 Participants

We aimed for a sample size of 28 participants. This is consistent with previous studies using the pattern classifier in the field of memory (Linde‐Domingo et al., 2019; Michelmann et al., 2016). Also, using the PANGEA analysis tool (https://jakewestfall.shinyapps.io/pangea/), 28 participants were judged to allow to detect the main effect and the three‐way interaction of medium effect size with a power exceeding 0.9. We also calculated, a posteriori, the power reached in the main effect of the memory we obtained in Experiment 1. All the participants for both experiments were recruited through the Research Participant Scheme of University of Birmingham for cash (£10/h) or course credits (1 credit/h). All of the participants had normal or corrected‐to‐normal vision. The eligibility criteria included native or excellent English proficiency, no history of neurological or psychiatric disorder, and having an experience of intense physical pain in their past. In order to ascertain that all the eligibility criteria were met, students who signed up for the study were contacted and screened before they were accepted as participants for the studies. During this initial screening phase, students were asked about their English proficiency, they had to complete a questionnaire where pathological history or psychotropic drugs assumptions were checked, and they had to complete the Autobiographical Memory Questionnaire (AMQ; Rubin, Schrauf, & Greenberg, 2003), for an experience of intense physical pain and for one emotionally and physically neutral that they were asked to report. Students who could not report any experience of intense physical pain or of a neutral experience could not be participants in these studies.

#### 2.1.1 Experiment 1. EEG

Thirty‐five healthy students took part in the experiment (mean age = 22, *SD* = 5). Four were left‐handed, six were males. Seven were discarded from the final sample, three were not Caucasian (we showed pictures of Caucasian faces and previous studies have shown that empathic responses are subject to ethnicity bias (e.g., Sheng & Han, 2012), two could not complete the task due to equipment failure and two for too low number of trials due to inaccurate responses. The final sample was composed of 28 participants (mean age = 21.96, *SD* = 4.82), four were males and four left‐handed.

#### 2.1.2 Experiment 2. fMRI

Thirty‐three healthy students took part in the experiment (mean age = 25, *SD* = 5.9). Participants were all right‐handed; 15 were males. Five participants were discarded from the final sample: two served as pilots to adjust the timing of the paradigm and make it suitable for the fMRI environment; one participant could not complete the acquisition session in the scanner, two were discarded for excessive movements (more than one voxel size, 3 mm). The final sample was composed of 28 participants (mean age = 24.71, *SD* = 5.86); 11 were males.

### 2.2 Questionnaires

As mentioned in Section 2.1, before accepting students as participants for the study, the students who signed up for the study underwent a screening phase. The screening consisted of collecting information about students' history of pathological morbidity, English proficiency and, critically, an AM of intense physical pain and of a neutral experience, in terms of emotion and pain. Candidates were therefore asked to report these AMs and complete the AMQ for both autobiographical episodes. The AMs reported by the participants were on average 4.65 years old (*SD* = 5 y) for Experiment 1; 4.46 years old (*SD* = 5.72 years) for Experiment 2.

At the end of the experimental session, dispositional empathy resources and the ability to recognize and describe participants' own emotions were assessed with the empathy quotient, EQ, and the interpersonal reactivity index (IRI; Baron‐Cohen & Wheelwright, 2004; Davis, 1983), and with the Toronto Alexithymia scale (TAS‐20; Bagby, Parker, & Taylor, 1994), respectively. Participants from both experiments fell in the normal range of the EQ (Experiment 1: *M* = 51.14, *SD* = 9.99; Experiment 2: *M* = 46.89, *SD* = 12.37), and had on average normal ability to describe their emotions as showed by the TAS score (Experiment 1: *M* = 45.96, *SD* = 12.22; Exp2: *M* = 43.96, *SD* = 12.01). The IRI scores for both experiments are reported in Table 1. These measures were also used to explore any relation with the neural responses, but no correlation was found significant (for further details on the correlation analysis, please see the Supplementary Materials).

**TABLE 1:** Participants' scores to the interpersonal reactivity index in both experiments. *t‐*Tests are performed on the two independent samples

	Exp 1		Exp 2		*t*(27)	*p*
	Mean	*SD*	Mean	*SD*		
F	3.89	0.89	3.81	0.71	0.569	>.5
Pt	3.74	0.75	3.73	0.81	0.067	>.5
EC	4.2	0.61	3.86	0.85	2.135	.042
Dp	2.92	0.75	2.85	0.74	0.51	>.5

### 2.3 Stimuli and procedure

All the stimuli were presented on a grey background of a 17″ computer screen with a refreshing rate of 70 Hz. The tasks were programmed using Psychtoolbox.

#### 2.3.1 Experiment 1: EEG

Participants performed two tasks in the same experimental session. For all participants, the first of these tasks was the empathy task used in previous studies (Meconi et al., 2018; Sessa, Meconi, & Han, 2014), and the second one was a retrieval task (Figure 1a,b).

##### The empathy task: Stimuli and procedure

The stimuli for the empathy task were sentences describing specific contexts, followed by faces, the task required to rate participants' empathy for the person as depicted in the preceding context. The faces were a set of 16 identities, 8 males and 8 females with a painful or neutral facial expression. The faces were in shades of grey, and they were equalized for luminance with the SHINE toolbox (Willenbockel et al., 2010). The sentences described contexts of a person feeling physical pain or depicted in an emotionally neutral context. The critical manipulation in this task was that the targets of participants' empathy were depicted in contexts for which they had or had not a related AM. Therefore, two contexts (one describing physical pain and the neutral one) were taken from participants' autobiographical experience. In order to tailor the contexts for each participant, we screened them prior to the experimental session as soon as they signed up for the study. Participants were asked to report an experience of intense physical pain and an emotionally and physically neutral experience for which they completed the AMQ. A physically painful and a neutral context that did not belong to participants' autobiographical experiences were also identified and used for the non‐AM contexts. The four contexts identified for each participant were described in the empathy task by a sentence that always followed the structure “This person got—[…]” or “This person did—[…]” so that all the sentences had the same syntactic complexity.

Each trial started with a fixation cross (600 ms). Participants were then presented with the sequence of a sentence (3 s) and a face (500 ms) interleaved by a variable fixation cross (800–1,600 ms jittered in steps of 100 ms). The task was to subjectively rate on a scale of six points how much empathy participants felt for the person as depicted in the presented context. The rate was self‐paced and presented after another fixation cross that was on the screen for 500 ms. At the end of the trial, participants were asked to indicate whether the face had a painful or neutral expression, regardless of the context. The task was composed of 48 trials per condition that were pseudo‐randomized so that the conditions were balanced over the whole session. There were 192 trials in total subdivided in 4 blocks. An illustration of the task is shown in Figure 1a.

##### The retrieval task: Stimuli and procedure

For the retrieval task, 16 shapes were created ad hoc in total. For each participant, a unique subset of 4 of these shapes was presented. The first step was to generate eight random polygons with equal number of black pixels. The polygons were then blurred with a Gaussian filter and all the pixels in shades of grey were made black to create eight rounded shapes. As a last step, the number of black pixels was equalized across all the shapes (i.e., random polygons and the rounded shapes). The shapes were shown on a grey background (Figure 1b). The sharp/rounded shapes were created leveraging the symbolic relationship that appears to occur between sounds and meanings, such as in Kohler's experiments involving the “bouba‐kiki” effect. In these experiments, participants consistently established meaningful correspondences between specific phonological values and rounded or angular shapes. We leveraged this effect to facilitate the association between the four contexts and the four cues: two sharp shapes for the painful contexts and two rounded shapes for the neutral contexts.

In the retrieval task, participants were required to picture the contexts described in the empathy task in their minds' eye. This task acted for the EEG pattern classifier as a localizer to extract the neural fingerprints of the contexts to then probe the data from the empathy task. Therefore, to avoid perceptual confounds when applying the classifier across tasks, participants were presented with their unique subset of four shapes that were used to cue the contexts described in the empathy task. Before starting the retrieval task, participants underwent a “learning phase” in which they learnt to associate each shape with one of the contexts, each sentence‐shape pair was presented twice before memory for the associations was tested. We let the participants memorize the associations with no time pressure. The retrieval task could only start when full accuracy was reached in the “learning phase.” To test memory for the sentence‐shape association, participants were presented only with a figure at a time and had to indicate what was the context associated with the shape. One cue‐word per sentence was chosen to cue to the related context and allow responses (e.g., we used “arm” as a cue‐word for the sentence “This person got their right arm broken”; “ligament” for “This person got their ligament torn”; “Museum” for “This person visited the Birmingham Museum of Art” and “laptop” for “This person bought a new laptop” and so forth). The four cue‐words were placed equally spaced horizontally at the centre of the screen and their order was randomized with a Latin square in such a way that each word had the same likelihood to appear at one of the four locations (e.g., “arm” “ligament” “museum” “laptop”; “museum” “arm” “laptop” “ligament” etc.). Participants could press one of four keys on the computer keyboard that spatially corresponded to the location of the cue‐word (“d” for the cue‐word appearing on the very left, “f” for the cue‐word appearing the central left, “j” for the one at the central right and “k” for the one at the very right location). The memory test could end after eight correct answers, that is, two times each pair. One error within a block of eight trials would be followed by the repetition of a new block of eight trials, until 100% accuracy would be reached. Once the memory associations test was successful participants could start the practice session of the retrieval task. Participants could familiarize with the retrieval task with a block of eight trials that could be repeated until they felt confident they understood the task.

In the retrieval task, participants were only shown the shapes. In each trial, one shape was shown for 3 s and participants had to picture in their mind's eye the context associated with that shape. Within the time the shape was on the screen, they were required to rate the vividness of the context as soon as they could picture it in their mind's eye, by pressing one of six response keys “s,” “d,” “f,” “j,” “k,” “l,” with “s” for “not vivid at all” to “l” for “very vivid.” If they did not press any button, a “No Response” was recorded and the trial excluded from the analysis. Participants were then asked to indicate which context they saw in their mind's eye. They could answer in the same way as they did for the memory association test with the further option that if they could not remember what context was associated with that shape, they could press the space bar for “forgotten” and move to the next trial. Responses were not time‐pressured but only correct trials were included in the analysis. Performance was consistently very high with no evidence of variation between conditions. We report this information in the Supplementary Materials. The task comprised 60 trials per condition that were pseudo‐randomized to balance the distribution of all the conditions over the 240 trials that constituted the whole session. The task, depicted in Figure 1b, right panel, was subdivided in four blocks.

#### 2.3.2 Experiment 2: fMRI

The screening phase, the questionnaires and the procedure were the same as those used in Experiment 1 with the exception of the necessary adjustments in the timing of the events applied to the empathy task in order to make it suitable for the fMRI environment (for additional details, see Supplementary Material S1.3.1).

### 2.4 Data acquisition and analysis

#### 2.4.1 Experiment 1: EEG

The EEG was recorded using a BioSemi Active‐Two system from 128 Ag/AgCl active electrodes. The EEG was re‐referenced offline to the average reference. Three additional external electrodes were placed below the left eye and on the lateral canthi of each eye to record vertical electroculogram (EOG). EEG and vertical EOG signals were digitized at a sampling rate of 1,024 Hz via ActiView recording software (BioSemi, Amsterdam, the Netherlands).

EEG data were analysed with MATLAB MathWorks, Munich, Germany) using the open‐source FieldTrip toolbox (http://fieldtrip.fcdonders.nl/) and in‐house MATLAB routines.

##### Pre‐processing

###### The empathy task

EEG data were first segmented into epochs of 2 s, starting 1 s before the onset of the face. The epoched data were visually inspected to discard large artefacts from further analysis. Further pre‐processing steps included Independent Component Analysis for ocular artefacts correction and re‐referencing to average reference. After removing trials which were contaminated by eye and muscle artefacts, an average of 45 trials (range: 34–48) remained for AM and 45 trials (range: 37–48) for non‐AM condition.

###### The retrieval task

EEG data were first segmented into epochs of 4 s, starting 1 s before the onset of the cue. The epoched data were visually inspected to discard large artefacts from further analysis. Further pre‐processing steps included Independent Component Analysis for ocular artefacts correction and re‐referencing to average reference. After removing trials which were contaminated by eye and muscle artefacts, an average of 51 trials (range: 42–60) remained for AM and 50 trials (range: 38–58) for non‐AM condition.

##### ERPs analysis: The empathy task

ERPs were time‐locked to the onset of the face. We computed ERPs in response to painful and neutral faces to test for replication of basic empathic response induced by painful faces as in previous studies in which we used this task (Meconi et al., 2018; Palmieri et al., 2021; Sessa, Meconi, & Han, 2014). To test any memory involvement in the empathy task, we contrasted ERPs time‐locked to the onset of the faces reflecting the processing of the preceding context (AM vs. non‐AM). To check whether there was any difference between the memories' emotional content, we also contrasted painful and neutral memories separately for AM and non‐AM.

##### LDA EEG pattern classifier

LDA is a multivariate pattern analysis method that finds a decision boundary that allows distinguishing the pattern of brain activity associated with one category of stimuli from the pattern of brain activity that is associated with another category of stimuli. This is based on specified features of the EEG signal. It can then estimate with certain accuracy whether the pattern of brain activity in data that was not used to find the decision boundary, is more similar to one or the other category of stimuli.

In order to reduce unwanted noise and computational time, the signal was filtered between 0.1 and 40 Hz and down sampled to 128 Hz before classification with a baseline correction window of 500 ms before the onset of the stimuli.

The LDA was trained and tested on the EEG raw patterns (i.e., amplitude of the signal on each of the 128 electrodes), for each participant and at each time point and regularized with shrinkage (Blankertz, Lemm, Treder, Haufe, & Müller, 2011; Treder, 2020).

To ensure that the output was not biased by the signal to noise ratio due to the different amount of trials, we equalized the number of trials for AM and non‐AM before training the classifier.

The classifier was trained on the raw signal (i.e., amplitude of EEG on each electrode) acquired while participants were performing the retrieval task in the time‐window including the presentation of the cue to detect systematic differences between the EEG patterns reflecting the representation of AM and non‐AM contexts. It was then tested on the signal independently acquired while participants were performing the preceding empathy task in the time‐window from the onset of the face until the rating was made. The aim of the LDA was to test for the online reactivation of the memory in preparation of the explicit judgement of participants' empathy awareness.

Before training and testing the LDA in two different datasets, we trained and tested the classifier on the retrieval task during the presentation of the cue to show that the task was successful to act as a localizer of the representation of the AM and non‐AM contexts. A K‐fold cross‐validation procedure with five repetitions was used to train and test the classifier. The output of this analysis is the accuracy with which the classifier could distinguish between the two memory contexts for each time‐point over all trials and electrodes. Therefore, the LDA reduces the data into a single decoding time course per dataset.

##### Source analysis

A standardized boundary element model was used for source modelling, which was derived from an averaged T1‐weighted MRI dataset (MNI, www.mni.mcgill.ca). That was used in combination with individual electrode positions. Individual electrodes' coordinates were logged with a Polhemus FASTRAK device (Colchester, VT) in combination with Brainstorm implemented in MATLAB 2014b (MathWorks). For three participants the standard electrode coordinates were used due to technical problems during the experimental session.

For source reconstruction, a time‐domain adaptive spatial filtering linear constrained minimum variance beamformer (van Veen, Drongelen, Yuchtman, & Suzuki, 1997), as implemented in fieldtrip was applied. Source analysis was carried out for the time‐domain ERP components that revealed significant results on the scalp level.

##### Statistical analysis

###### Behaviour: the Empathy Task

Mean proportions of accurate responses given within ±2.5 *SD* from the average reaction time of each participant and mean proportions of the empathy awareness scores were computed for each condition and inserted in two repeated‐measures ANOVAs with a 2 (Emotional memory: Painful vs. Neutral) × 2 (Memory: Autobiographical vs. non‐Autobiographical) × 2 (Facial expression: Painful vs. Neutral) as within‐subject factors. Bonferroni corrected paired‐sample *t* tests were conducted when appropriate to explore significant interactive effects. Partial eta squared (*ƞ*_*p*_^2^) are reported for completeness and transparency. Effect sizes are reported as eta squared (*ƞ* ^2^) calculated as the ratio between the sum of squares of each effect and the sum of the sum of squares of all the effects and their errors, 95% confidence intervals (CI) of the mean differences between conditions are reported in squared brackets.

###### ERPs—Empathy task

Cluster‐based permutation tests were performed over the whole scalp and over a 1 s time‐window on the event‐related potentials time‐locked to the onset of the face. We tested for significant differences between painful and neutral facial expressions in order to replicate previous findings and show an ERP empathic response to faces. Additionally, preliminary analysis was carried out to test for any involvement of memory in the pain decision task and whether there was any difference related to the emotional content of the memory. To this end, cluster‐based permutation tests were performed on the ERPs time‐locked to the onset of the face regardless of the facial expression contrasting AM versus non‐AM and painful and neutral contexts separately for AM and non‐AM.

###### ERPs—Retrieval task

Cluster‐based permutation tests were performed over the whole scalp and over the time‐window of cues presentation on the event‐related potentials time‐locked to the onset of the cue. Quality of data was checked and analysis was carried out to test for any preliminary difference related to the type of memory.

###### LDA classifier

For the classifier analysis, an empirical null distribution was created with a combined permutation and bootstrapping approach (Stelzer, Chen, & Turner, 2013) that tested whether the maximum cluster of accuracy values above the chance level was statistically significant. Clusters were identified on the basis of the number of adjacent pixels found with the MATLAB function bwlabel. We used the LDA in 100 matrices with pseudo‐randomly shuffled labels independently for each participant and created a null distribution of accuracy values that we contrasted with the LDA outputs obtained with the real data. This was done by sampling with replacement 100,000 times from the real and random data of each subject and computing a group average. This procedure resulted in an empirical chance distribution, which allowed us to investigate whether the results from the real‐labels classification had a low probability of being obtained due to chance (*p* < .05) (i.e., exceeding the 95th percentile).

#### 2.4.2 Experiment 2. fMRI

Data acquisition was performed with 3 T Philips Medical Systems Achiva MRI scanner using a 32‐channel head coil. Functional T2‐weighted images were acquired with isotropic voxels of 3 mm, repetition time (TR) = 1,750 ms, echo time (TE) = 30 ms, field of view (FOV) = 240 × 240 × 123 mm, and flip angle = 78°. Each volume comprised 33 sequentially ascending axial slices with an interslice gap of 0.75 mm). Each participant underwent four blocks of scan series; one full block comprised 410 volumes. A high‐resolution T1‐weighted anatomical scan was acquired with an MPRAGE sequence (TR = 7.4 ms, TE = 3.5 ms, isotropic voxel size of 1 mm, FOV = 256 × 256 × 176, flip angle = 7°) after the first two functional scanning blocks. The MR scanner was allowed to reach a steady state by discarding the first three volumes in each of the four scan series block.

##### Pre‐processing

The analyses were performed using the SPM12 toolbox (University College London, London, UK; http://www.fil.ion.ucl.ac.uk/spm/). For each scanning block, a motion realignment of each slice to the first slice was carried out before time realignment (slices corrected to the middle one). Data was then linearly detrended, using a Linear Model of Global Signal algorithm (Macey, Macey, Kumar, & Harper, 2004) to remove any minimal fluctuation due to the physical setting. Functional images served as reference for the co‐registration with the anatomical image. The data were further normalized to an MNI template, and finally, images were spatially smoothed with an 8‐mm FWHM Gaussian kernel.

##### Whole‐brain analysis

Two separate univariate analyses were carried out for two different time‐windows, one analysis was time‐locked to the onset of the face, and the other was time‐locked to the onset of the context. This was only done to parallel Experiment 1 and not to test for any functional dissociation between the two time‐windows. In both cases, statistical parametric maps were created for each participant's block of trials.

AM and non‐AM conditions were directly contrasted in paired‐sample *t* tests on a group‐level analysis.

The first analysed fMRI data were time‐locked to the onset of the context. Regressors were defined for AM and non‐AM related to the onset of the contexts regardless of the emotional content of the context described by the sentence. Additional regressors of no interest were again included in the design matrix to explain variance in the data not due to the experimental manipulation under investigation and the six motion parameters obtained during the realignment phase of the pre‐processing. Sixty statistical parametric maps were created (4 blocks × 15 regressors) for each participant.

The second analysed fMRI data were time‐locked to the onset of the face. Regressors were defined for autobiographical and non‐AM time‐locked to the onset of the faces regardless of their emotional expression. Additional regressors of no interest were included in the design matrix to explain variance in the data not due to the experimental manipulation under investigation plus the six motion parameters. Fifty‐four statistical parametric maps were created (4 blocks × 14 regressors) for each participant. Additional information on the regressors of no interest are reported in Supplementary Material S1.3.2.2.

##### Statistical analysis

###### Behaviour

Mean proportions of the empathy awareness scores were computed for each condition and inserted into a repeated measures ANOVA with a 2 (Emotional memory: Painful vs. Neutral) × 2 (Memory: Autobiographical vs. non‐Autobiographical) × 2 (Facial expression: Painful vs. Neutral) as within‐subject factors. Bonferroni corrected paired‐sample *t* tests were conducted when appropriate to explore significant interactive effects. Partial eta squared is reported for completeness and transparency. Effect sizes are reported as eta squared (*ƞ* ^2^) calculated as the ratio between the sum of squares of each effect, and the sum of the sum of squares of all the effects and their errors, 95% CI of the mean differences between conditions are reported in squared brackets.

###### fMRI

For both time‐windows, a within‐subject analysis was carried out on the data set of each participant to obtain the mean statistical parametric map for each experimental condition. Finally, a group‐level paired‐sample *t* test contrasting AM and non‐AM was performed. A cluster‐wise analysis was performed with uncorrected *p* = .001 and then Family Wise Error correction was applied for multiple comparison (cluster *p* threshold = .05). Peak voxel MNI are reported in brackets. Further information on the fMRI analysis and results can be found in the Supplementary Materials (S1.3.2.1 and S2.2.1).

## 3 RESULTS

### 3.1 Behavioural results

#### 3.1.1 Experiment 1: EEG

Individual scores of the empathy awareness revealed a main effect of the type of memory *F*(1,27) = 22.319; *p* = .000064, *η*_*p*_^2^ = .453, *η* ^2^ = .092, *M*_diff_ = .767 CI_95_ = [.434 1.10] such that AM context induced higher empathy rates than non‐AM contexts; of the emotional content of the memory *F*(1,27) = 50.902; *p* < .000001, *η*_*p*_^2^ = .653, *η* ^2^ = .157, *M*_diff_ = 1.0, CI_95_ = [−1.288–.713] and of the facial expression *F*(1,27) = 42.270; *p* < .000001, *η*_*p*_^2^ = .613, *η* ^2^ = .193, *M*_diff_ = 1.108, CI_95_ = [−1.456–.760] such that painful conditions induced higher rates than neutral conditions. The two‐way interaction between emotional content of the memory and of the facial expression was significant *F*(1,27) = 18.390; *p* = .000206, *η*_*p*_^2^ = .405, *η* ^2^ = .08. Further exploration of the two‐way interaction revealed that painful faces drove higher rates of empathy awareness than neutral faces when the emotional content of the preceding memory was painful (*t*(27) = 9.7, *p*_*c*_ < .0000001, *M*_diff_ = 1.821, CI_95_ = [1.436 2.207]) but not when it was neutral (*p*_*c*_ = .167). In the same vein, painful memories reported higher empathy awareness scores than neutral memories when followed by painful (*t*(27) = 10.825, *p*_*c*_ < .0000001, *M*_diff_ = 1.714, CI_95_ = [1.389 2.038]) but not neutral faces (*p*_*c*_ = .286). The three‐way interaction between the three factors was also significant *F*(1,27) = 11.002; *p* = .003, *η*_*p*_^2^ = .290, *η* ^2^ = .003 such that empathy rates for painful faces was higher for painful when compared to neutral contexts for both AM (*t*(27) = 8.219, *p*_*c*_ < .0001, *M*_diff_ = 1.927, CI_95_ = [1.165 2.689]) and non‐AM (*t*(27) = 6.397, *p*_*c*_ < .0001, *M*_diff_ = 1.500, CI_95_ = [.738 2.262]) but this difference was higher for AM than non‐AM contexts (*t*(27) = 2.122, *p* = .043, *M*_diff_ = .427, CI_95_ = [.014 .840]). However, this result was not robust enough (power reached: 66%).

We further explored the three‐way interaction by conducting separate ANOVAs for painful and neutral faces. We only observed a main effect of memory *F*(1,27) = 20.917; *p* < .001, *η*_*p*_^2^ = .437, *η* ^2^ = 180, for neutral faces such that for both emotional contents of the memories, AM contexts drove higher empathy rates than non‐AM contexts. For painful faces, we observed the main effect of memory *F*(1,27) = 21.429; *p* < .001, *η*_*p*_^2^ = .442, *η* ^2^ = .104, and the main effect of emotional content of the memory *F*(1,27) = 117.175; *p* < .001, *η*_*p*_^2^ = .813, *η* ^2^ = .571, and the two‐way interaction *F*(1,27) = 4.502; *p* = .043, *η*_*p*_^2^ = .143, *η* ^2^ = .009. Also in this case, for both emotional contents of the memories, AM contexts drove higher empathy rates than non‐AM contexts (minimum Bonferroni corrected *t*(27) = 2.69 *p* = .012, *d* = .51, power reached 83%).

We also conducted separate ANOVAs for emotional content of the memories. For neutral memories, we observed the main effect of memory *F*(1,27) = 13.788; *p* = .001, *η*_*p*_^2^ = .338, *η* ^2^ = .127, but not of the face *F*(1,27) = 2.013; *p* = .167, *η*_*p*_^2^ = .069, *η* ^2^ = .042. We also did observe the two‐way interaction between face and memory *F*(1,27) = 14.991; *p* = .001, *η*_*p*_^2^ = .357, *η* ^2^ = .008. For both neutral and painful faces, we observed higher empathy rates in AM than non‐AM contexts (minimum *t*(27) = 3.913; *p* = .001, *d* = .74, power reached: 98%). For neutral memories followed by either neutral or painful faces, AM drove higher empathy rates than non‐AM.

For painful memories, we observed a main effect of face *F*(1,27) = 94.083; *p* < .001, *η*_*p*_^2^ = .777, *η* ^2^ = .561 and of memory *F*(1,27) = 23.737; *p* < .001, *η*_*p*_^2^ = .468, *η* ^2^ = .120, but no two‐way interaction *F*(1,27) = 2.344; *p* = .137, *η*_*p*_^2^ = .080, *η* ^2^ = .002. For both neutral and painful faces we observed higher empathy rates in AM than non‐AM contexts (minimum *t*(27) = 2.687; *p* = .012, *d* = .74, power reached: 83%).

Summarizing the set of Bonferroni corrected comparisons, we observed higher empathy rates for painful than neutral emotional memories followed by painful faces when compared to when contexts were followed by neutral faces, both for AM and non‐AM contexts. We then computed the differential scores of the empathy rates for painful and neutral faces and for painful and neutral memories and contrasted these differences between AM and non‐AM contexts. Increased empathy rates obtained for painful memories followed by painful faces was bigger for AM than non‐AM contexts *t*(27) = 3.317; *p* = .003, *d* = .63 (power reached: 94%).

#### 3.1.2 Experiment 2: fMRI

Individual scores of the empathy awareness revealed a main effect of the type of memory *F*(1,27) = 7.210; *p* = .012, *η*_*p*_^2^ = .211, *η* ^2^ = .021, *M*_diff_ = .355, CI_95_ = [.084 .626] (reaching 82% of power and therefore providing good replication of the behavioural result observed in Experiment 1); of the emotional content of the memory *F*(1,27) = 48.860; *p* < .000001, *η*_*p*_^2^ = .644, *η* ^2^ = 0.186, *M*_diff_ = 1.064, CI_95_ = [.752 1.376] and of the facial expression *F*(1,27) = 38.863; *p* = .000001, *η*_*p*_^2^ = .590, *η* ^2^ = 0.186, *M*_diff_ = 1.065, CI_95_ = [.714 1.415]. The two‐way interaction between emotional content of the memory and of the facial expression was significant *F*(1,27) = 19.995; *p* = .000126, *η*_*p*_^2^ = .405, *η* ^2^ = .096, so was the one between the emotional content and the type of memory *F*(1,27) = 4.758; *p* = .038, *η*_*p*_^2^ = .150, *η* ^2^ = .008. Further exploration of the two‐way interactions revealed that painful faces drove higher rates of empathy awareness than neutral faces when the emotional content of the preceding memory was painful (*t*(27) = 8.8, *p*_*c*_ < .0000001, *M*_diff_ = 1.83, CI_95_ = [1.404 2.257]) but not when it was neutral (*p*_*c*_ = .280). In the same vein, AM reported higher empathy awareness scores than non‐AM when they were painful (*t*(27) = 3.016, *p*_*c*_ = .006, *M*_diff_ = .578, CI_95_ = [.185 .971]) but not when they were neutral (*p*_*c*_ = .348). However, painful memories reported higher scores of empathy than neutral memories, were they either autobiographical or not (min *t*(27) = 5.17, *p*_*c*_ = .00002, *M*_diff_ = .841, CI_95_ = [.507 .1.175]). The three‐way interaction did not reach significance level *F*(1,27) = 2.294; *p* = .142, *η*_*p*_^2^ = .078, *η* ^2^ = .0006.

The behavioural results from the two experiments showed that individuals depicted in contexts describing participants' autobiographical contexts drove enhanced explicit judgements of empathy awareness when compared to contexts describing non‐autobiographical contexts independently of all the other factors. These results are shown in Figure 1c).

### 3.2 EEG results

#### 3.2.1 ERPs and source analysis

**FIGURE 2:**
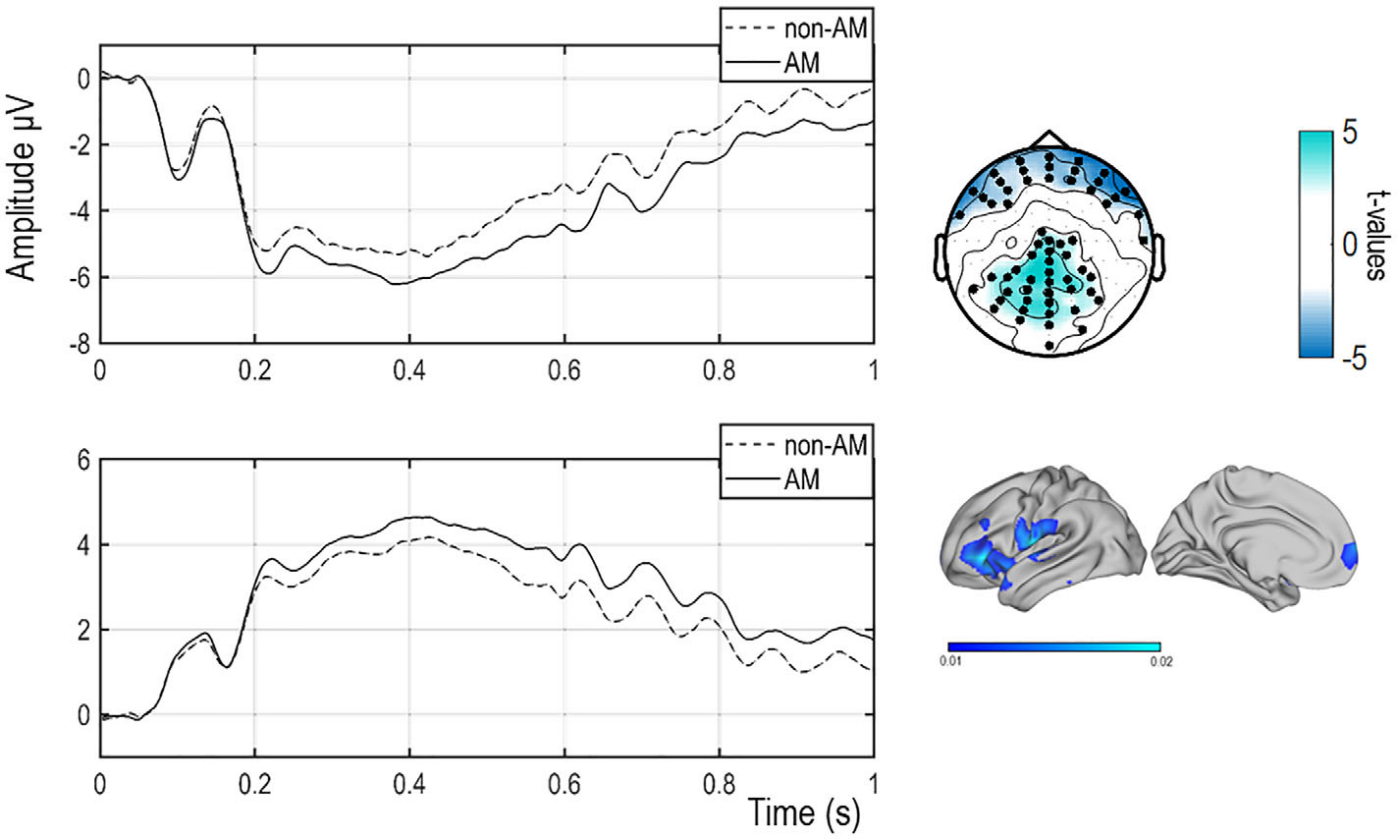
Event‐related potentials (ERPs) results. Left panel: ERPs time‐locked to the onset of the face and reflecting autobiographical memories (AM) and non‐AM at the anterior (upper panel) and the posterior cluster (bottom panel. Right top panel: clusters analysis performed over all the electrodes in a 0–1 s time‐window. Colours code *t*‐values. Right bottom panel: source localization of the AM versus non‐AM contrast

Cluster analysis conducted over a 1 s time‐window, from the onset of the face until the presentation of the rating, revealed one anterior and one posterior cluster of electrodes showing that ERPs significantly differ as a function of the type of memory (anterior: *p* = .002; posterior: *p* = .002). Figure 2 depicts ERPs for AM and non‐AM in the left panel and the topography of the significant clusters in right upper panel, *t*‐values are plotted). Source analysis estimated that the neural source of this effect was the superior frontal gyrus, BA 10, MNI: [−10 69 0] (Figure 2 right bottom panel). No difference was found for separate contrasts between emotional contents of the context for neither AM (*p* = .06) nor for non‐AM (*p* = .18), therefore we did not further analyse differences between emotional contents of the contexts.

Additionally, in line with previous studies on empathy for physical pain, cluster analysis also revealed a classic ERP response associated with empathic processes (e.g., Sessa, Meconi, Castelli, & Dell'Acqua, 2014; Sessa, Meconi, & Han, 2014), that is, painful faces elicited more positive ERP responses than neutral faces (*p* = .004). Consistently, source analysis estimated that the neural source of this effect was the inferior frontal gyrus, BA 9, MNI: [−62 21 30] and the parietal lobule, BA 7, MNI [30–69 48].

As for the retrieval task, cluster analysis conducted over a 1 s time‐window, between 1 and 2 s from the onset of the cue, revealed one left central cluster of electrodes showing that ERPs significantly differ as a function of the type of memory (*p* = .044). A significant cluster of electrodes was also observed between ERPs reflecting painful AM and non‐AM contexts (*p* = .03) and a similar result was observed for neutral AM versus non‐AM contexts (*p* = .013). Graphical representation of these results is reported in the Supplementary material, Figure S1.

#### 3.2.2 Linear discriminant analysis

We first ran a sanity check of the classifier on the retrieval task. The classifier was trained and tested with a K‐fold cross‐validation procedure during the presentation of the cue (Figure 1b) as reported in LDA EEG pattern classifier section. Since we were interested in investigating whether AM is reactivated in empathy, we checked that the classifier could distinguish first of all whether the context pictured in the participants' mind's eye was an AM or a non‐AM.

**FIGURE 3:**
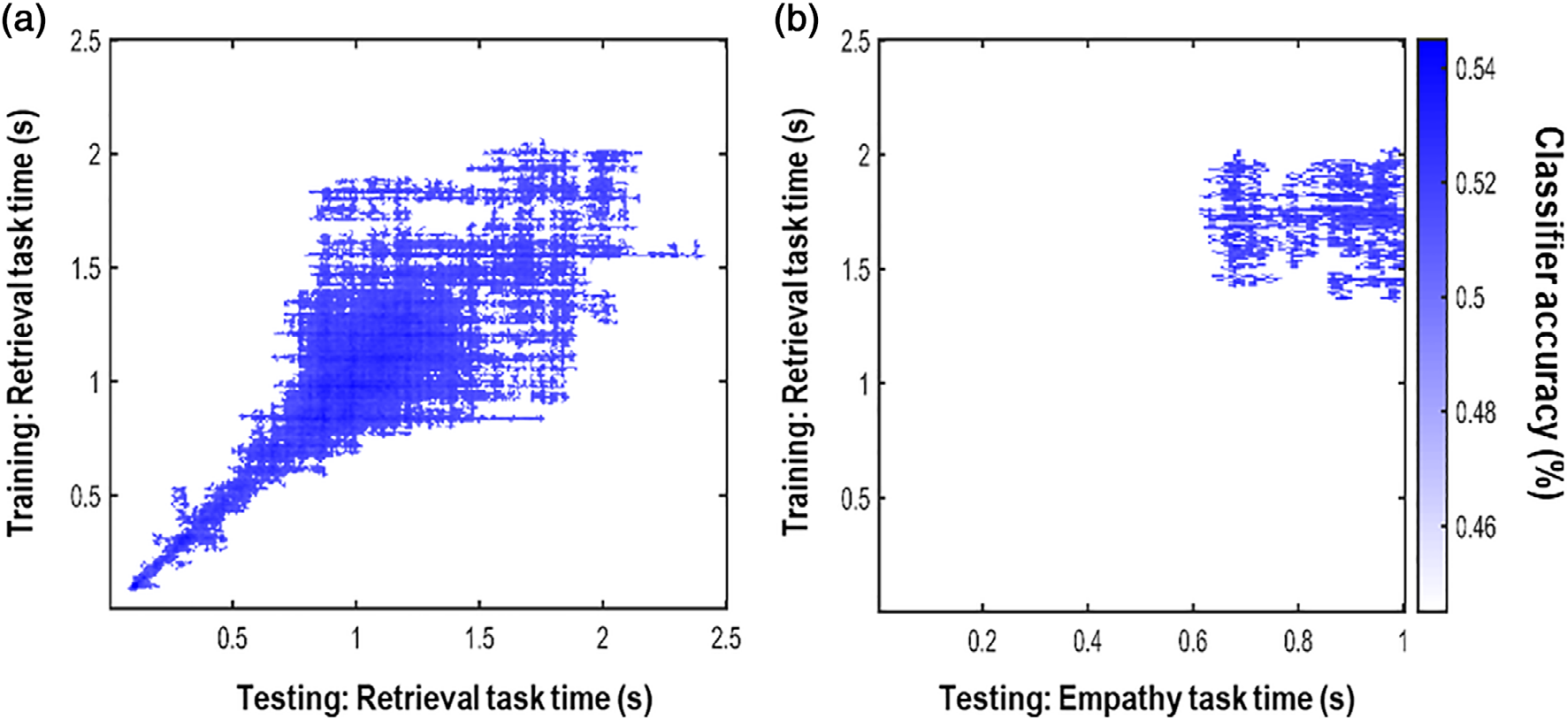
Linear discriminant analysis (LDA) results (a) Sanity check: time by time generalization matrix showing significant classification of autobiographical memories (AM) versus non‐Am within the retrieval task. (b) Time by time generalization matrix (i.e., training and testing at each time‐point) showing significant classification of AM versus non‐AM across tasks

The square shape of the time by time generalization matrix shown in Figure 3a showed that the task allowed the formation of stable representations associated with the figures (one random polygon and one rounded shape for AMs and the same for the non‐AMs) acting successfully as a localizer for the two types of memories. The bootstrapping analysis performed on a 0–2.5 s time‐window showed that the classifier could distinguish with a peak accuracy of 0.55 between AM and non‐AM (*p* = .0129) in a sustained time‐window (0–~2.2 s), including a late time window that is most likely related to the representation of the memory itself rather than to any perceptual features of the stimuli. In a second step, the classifier was trained during the presentation of the cue in the retrieval task and then tested on the pain decision task in a 1 s time‐window starting from the onset of the face. Crucially, any consistency in the neural pattern observed across tasks would show the representation of the memories. The bootstrapping analysis revealed a significant cluster (*p* = .032) in a sustained time‐window (0.6–1 s) showing evidence for the online reactivation of the memory in preparation of the empathy judgement with a peak accuracy of 0.53. Although the accuracy seems modest, the significant cluster demonstrates consistent information was processed across tasks and it is in line with other studies in which decoding was performed in a retrieval stage of memory processes (Hebart & Baker, 2018; Kerrén, Linde‐Domingo, Hanslmayr, & Wimber, 2018; Kurth‐Nelson, Barnes, Sejdinovic, Dolan, & Dayan, 2015). The result of the classifier across tasks is shown in Figure 3b.

### 3.3 fMRI results

**FIGURE 4:**
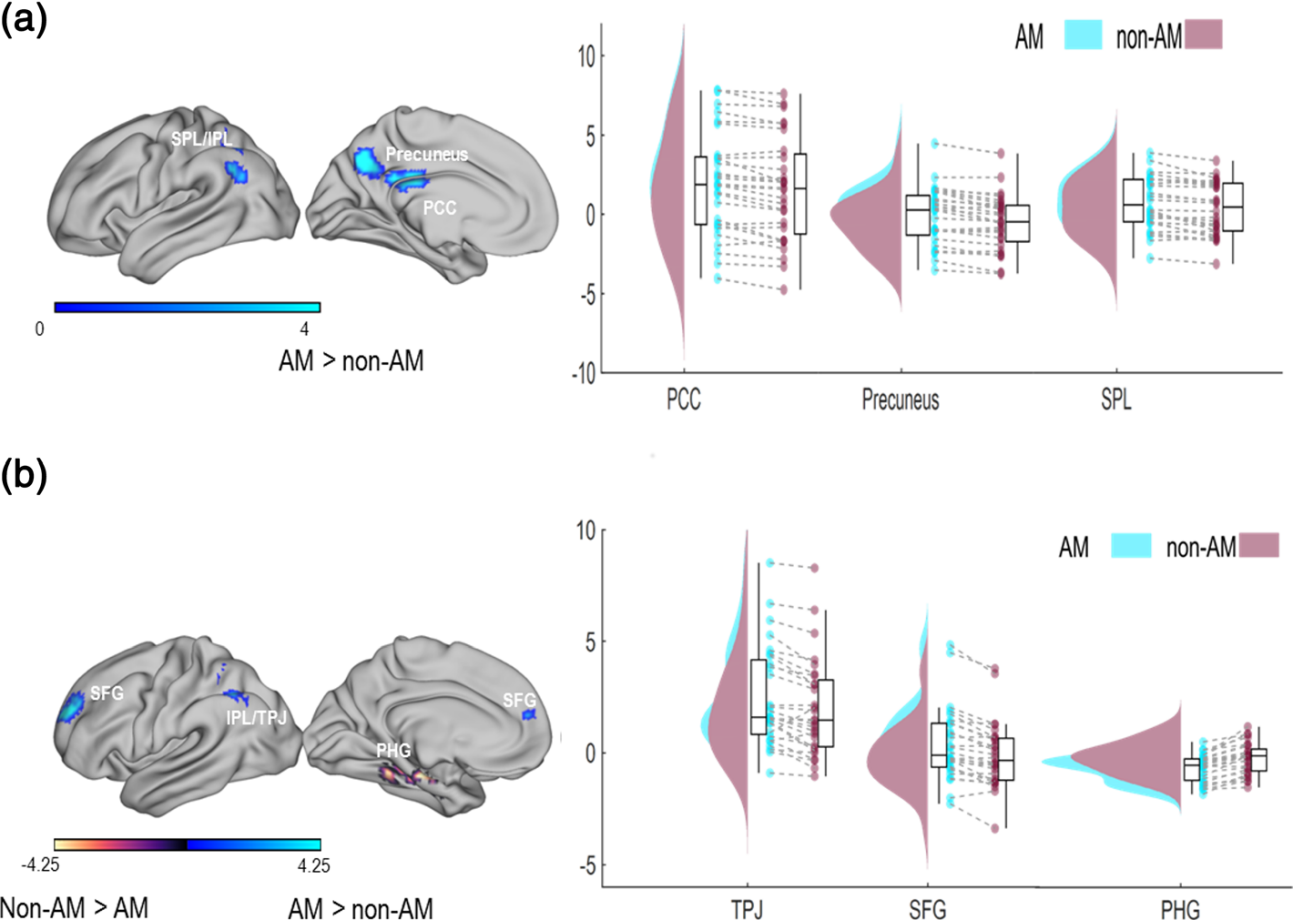
fMRI results. whole‐brain analysis results (left panel) and raincloud plots (right panel) of the activation in each condition and each cluster. (a) Whole‐brain analysis related to the presentation of the context. Only the contrast autobiographical memories (AM) > non‐AM showed significant clusters. (b) Whole‐brain analysis related to the presentation of the face. Figure shows significant clusters resulting from both the AM > non‐AM and non‐AM > AM contrasts

Figure 4 shows masked clusters resulting from the whole‐brain analysis.

The contrast AM > non‐AM for the analysis time‐locked to the onset of the context revealed a significant FWE corrected (*p* < .05) cluster with a peak in the precuneus, BA 7, MNI: [3 −64 38], (150 voxels), *t*(27) = 5.76, *p* = .001, in the superior parietal lobule, BA 7, MNI: [−36 −58 59], (114 voxels), *t*(27) = 4.42, *p* = .003 extending to the inferior parietal lobule (BA 40) and in the superior temporal gyrus (BA 39). This contrast also revealed a cluster with a peak in the posterior cingulate, BA 23, MNI: [3–28 26], (62 voxels), *t*(27) = 4.71, *p* = .038. Masked clusters showing greater activation for AM as compared to non‐AM are depicted in Figure 4a. The opposite contrast did not reveal any significant FWE corrected cluster.

Figure 4b shows the result of the contrast AM > non‐AM for the analysis time‐locked to the onset of the face. Greater activation for AM as compared to non‐AM was observed in a significant FWE corrected cluster (*p* < .05) in the superior frontal gyrus, BA 10, MNI: [−18 62 23], (66 voxels), *t*(27) = 5.49 *p* = .024, and in a cluster in the inferior parietal lobule, BA 39, MNI: [−36 61 41], (75 voxels), *t*(27) = 3.67, *p* = .014. This specific region of the IPL is part of the functional fractionation of the TPJ and is considered as part of the core network of the theory of mind (Schurz, Radua, Aichhorn, Richlan, & Perner, 2014). The opposite contrast revealed greater activation for non‐AM than AM in a significant FWE corrected cluster in the parahippocampal gyrus (PHG), BA 36, MNI: [−18 −16 −22], (169 voxels), *t*(27) = 6.22, *p* < .001.

We performed six correlational analyses between differential hemodynamic activity in each of these significant clusters and individual differential empathy judgements for AM versus non‐AM contrasts. We would only accept *p*‐values <.0083. We observed significant correlation between activation in the SFG and the differential empathy judgements *r* = .423, *p* = .025, which did not survive multiple comparisons correction; we also found a robust correlation between the activation in the PHG and the differential empathy judgements *r* = −.661, *p* = .0001. Scatterplots for these correlations are reported in the Supplementary Materials.

## 4 DISCUSSION

In the current study, we recorded EEG and hemodynamic activity from two independent samples of participants to investigate whether AMs are reactivated in the service of empathy. The present results from two independent experiments show consistent behavioural, electrophysiological, and fMRI evidence supporting a direct engagement of AM reactivation in empathy. Our experiments provide insights into the mechanism implied by previous studies suggesting that participants' past experiences interact with cognitive empathic abilities (Bluck et al., 2013; Gaesser & Schacter, 2014; Perry et al., 2011).

In Experiment 1, EEG was recorded during a task that prompted empathy, and a memory retrieval task. First of all, we showed that the empathy task‐induced basic empathic response to painful faces as in previous studies in which this task has been adopted (Meconi et al., 2018; Palmieri et al., 2021; Sessa, Meconi, & Han, 2014). Cortical EEG patterns during the retrieval task were used to probe the data from the empathy task for evidence of reactivation of AMs and non‐AMs. We applied an LDA classifier trained and tested across tasks and found evidence for online memory reactivation when participants explicitly judged their empathy for others' experiences. Participants could empathize more with people depicted in situations they had experienced themselves as compared to situations that they never experienced, as reflected in self‐reported rates of participants' empathy awareness. This behavioural result was replicated in Experiment 2 showing the robustness of this behavioural evidence.

Three critical features of the study design underwrite the robustness of our findings. First, the autobiographical component of the memories used to probe empathy was unprompted in the empathy task. Participants' AM retrieval could have no impact on the rating of their empathy awareness unless participants based their judgement on their own past experience. Therefore, the EEG evidence for reactivation of AM patterns is remarkable because participants could have relied entirely on their semantic knowledge to perform the tasks, yet the above chance performance of the classifier suggests that they did not. Second, the memory retrieval task was always performed after the empathy task to avoid that participants could be primed to retrieve their own memories. Third, we used perceptually different stimuli to prompt empathy for specific contexts (sentences) and trigger those episodes' reactivation (shapes). This was done to avoid any overlap in the perceptual features that cued the memories in the two tasks and ensure that the classifier could only identify the neural fingerprint of the memories' reactivation per se. Our results suggest that memory retrieval and empathic processes operate within the same time‐frames. The EEG pattern classifier approach has been successfully adopted to differentiate between the retrieval of the perceptual and semantic content of an episodic memory (Linde‐Domingo et al., 2019) and in different mechanisms of memory (Jafarpour, Horner, Fuentemilla, Penny, & Duzel, 2013). The timing of the retrieval of an AM has been shown to occur between 400 and 600 ms even when it is only spontaneously recalled (Addante, 2015; Hebscher, Ibrahim, & Gilboa, 2020). The squared shape of the time by time generalization output depicted in Figure 3 shows that the representation is stable across time (King & Dehaene, 2014). Figure 3a shows that the representation of the memories starts stabilizing between 500 ms and 1 s and lasts until ~2 s. Figure 3b shows that the representation of the memories reactivated in the time window when the empathy judgement was prepared.

In Experiment 2, we recorded fMRI in an independent sample of participants performing the same empathy task as in Experiment 1 with the purpose to replicate behavioural findings verify in and broaden the picture of neural engagement of AM in empathic processes. We tested whether AM would show increased activation when compared to non‐AM contexts in those frontoparietal brain areas that have robustly been associated with the cognitive empathy network (Lamm et al., 2011; Molenberghs et al., 2016). This second experiment replicated the behavioural result obtained in Experiment 1: participants reported increased empathy awareness for individuals described in contexts for which they had an associated AM. Whole‐brain analyses contrasting the hemodynamic response for AM and non‐AM were conducted related to the onset of the context, that is, when participants read the sentences describing either an AM or a non‐AM, and of the face, conveying painful or neutral expression. The first analysis showed activation of the precuneus (BA 7), PCC (BA 23), and left SPL (BA 7). The second analysis activated the left SFG (BA 10) and a specific region of the left IPL, part of the functional fractionation of the TPJ (BA 39). The activation of these brain areas is consistent with previous literature showing that these brain areas underlie cognitive empathic processes (Amodio & Frith, 2006; Bernhardt & Singer, 2012; Buckner & Carroll, 2007; Frith & Frith, 2003; Spreng et al., 2008; Zaki & Ochsner, 2012).

The parietal cortex is a critical hub for cognitive empathic processes, and AM retrieval. The SPL is involved in the online maintenance of relevant information (Postle, Awh, Jonides, Smith, & D'Esposito, 2004; Xie, Li, Xie, Xu, & Peng, 2019) and in the retrieval of specific AM (Addis, McIntosh, Moscovitch, Crawley, & McAndrews, 2004). A recent study by Hebscher et al. demonstrated the causal involvement of the precuneus in AM retrieval (Hebscher et al., 2020). The involvement of the parietal cortex in the retrieval of AM, and in particular of the precuneus, has been suggested to be responsible of the spontaneous AM retrieval from an egocentric perspective (Freton et al., 2014) and in flexible perspective shifting during AM retrieval (St. Jacques, Szpunar, & Schacter, 2017). Therefore, it ultimately contributes to the vividness of the retrieval and of constructing realistic mental images (Fuentemilla, Barnes, Düzel, & Levine, 2014). The precuneus is reliably engaged in the network of brain areas underlying the understanding of others' mind, that is, cognitive empathy (Molenberghs et al., 2016). The PCC is involved in the retrieval of familiar objects and places (Burianova & Grady, 2007) and, together with at least the anterior division of the precuneus, in self‐referential processes (Sajonz et al., 2010).

In the whole‐brain analysis contrasting the AM and non‐AM related to the onset of the face, we observed the activation of a specific region of the left IPL, part of the functional fractionation of the TPJ (BA 39) and of the left SFG (BA 10). A recent meta‐analysis investigating the core network of theory of mind (Schurz et al., 2014) demonstrated that, together with the mPFC, the left TPJ is a core brain area of this network (Gaesser, Hirschfeld‐Kroen, Wasserman, Horn, & Young, 2019). Lesion studies further support this view as damage of the left TPJ can selectively reduce theory of mind abilities but no other cognitive or executive abilities (Apperly, Samson, Chiavarino, & Humphreys, 2004; Bzdok et al., 2013; Samson et al., 2004). The activation of the left SFG was in line with the source estimation of the ERP data in Experiment 1 for the same contrast and time window. We also observed a significant correlation between this increased activation for AM versus non‐AM and differential empathy judgements. This correlation did not survive multiple comparisons correction, but it is worth noticing that this result is in line with further replication of our findings from both experiments. ERP studies investigating empathy for physical pain have shown that an empathic reaction, reflecting the processing of a painful experience, is expressed as a positive shift of the ERP response, compared to a neutral condition with (e.g., Meconi et al., 2018; Sessa & Meconi, 2015) or without (Sheng & Han, 2012) relation to explicit or implicit measures of empathy. In Experiment 1, we observed a positive shift in the ERPs reflecting the processing of painful when compared to neutral faces within 1 s in a cluster of centroparietal electrodes that was estimated to be generated in the IPL and the IFG. Within the same time window, ERPs time‐locked to the onset of the faces reflecting the processing of the preceding memory showed a positive shift of the ERPs for AM compared to non‐AM. The neural source of this effect was estimated to be in the SFG. According to the multiple memory system of social cognition, prejudice and stereotyping are the result of affective and semantic associations in memory (Amodio & Ratner, 2011) resulting from autobiographical experience and acquired knowledge. Studies on cross‐racial empathy for pain showed that empathic responses are more natural for own‐race faces or more familiar faces when compared to other‐race faces (Avenanti, Sirigu, & Aglioti, 2010; Sessa, Meconi, Castelli, & Dell'Acqua, 2014; Xu, Zuo, Wang, & Han, 2009). Therefore, these ERP studies provided some parallel evidence that past experiences and shared cultural background can influence empathy as they contribute to reducing the psychological distance between the observer and the target of empathy (Meconi, Vaes, & Sessa, 2015). Our ERP results and the source analysis for the face and memory effects are in line with our fMRI results obtained in Experiment 2; furthermore, these findings replicate previous ERP (Sessa, Meconi, & Han, 2014) and neuroimaging studies on the neural correlates of cognitive empathy (Bernhardt & Singer, 2012; Fan, Duncan, de Greck, & Northoff, 2011; Lamm et al., 2011). However, it is important to mention that the area obtained from the source reconstruction in Experiment 1, that is, the SFG, seems not to fully overlap with the one observed in Experiment 2 from the fMRI. It is possible that the wider cluster obtained in Experiment 1 could have been due to lower precision in the source reconstruction of an ERP effect compared with source localization from the fMRI data. Equally, though it is possible that the MNI coordinates in the fMRI analysis only identify the peaks of the clusters that in fact reflect the activation of the same, larger, brain area identified from the ERP data.

In Experiment 2, we did not observe the activation of the MTL in the contrast AM > non‐AM. Notably, the participants' AMs were on average 5 years old. A recent review (Barry & Maguire, 2019) highlighted that although memories seem to become independent from hippocampal activation with remoteness in time, the hippocampus remains involved in context/memory reconstruction (Zeidman & Maguire, 2016) even though the original memory trace is with time transferred to the neocortex. We did observe the activation of the PHG in the contrast non‐AM > AM. This result strongly correlated with individual differential empathy judgements between AM and non‐AM contexts; it nicely dovetails with the mindreading hypothesis (Gaesser, 2018) that draws on those studies with healthy participants showing the involvement of the episodic simulation in performing tasks that prompt cognitive empathy. Consistently, patients with MTL lesions do not show increases in empathy when prompted to use episodic simulation to construct specific episodes of others suffering (Sawczak, McAndrews, Gaesser, & Moscovitch, 2019).

In the current research, we did not observe compelling results regarding the specificity of the painful content of the memories so that painful AM robustly showed enhanced empathy when compared to painful episodes for which participants did not have associated any AM. Namely, although painful memories increased empathy rates, we did not observe exceptional empathy effects on neural correlates for the painful AMs. Noteworthy, a set of recent neuroimaging studies investigated empathy for pain involving direct referral to the personal experience, that is, first‐hand pain or touch. In these studies, participants either observed a confederate receiving a painful shock or being touched and received a painful shock or were touched first‐hand. By experimentally inducing first‐hand experience, these studies explicitly investigated the affect sharing aspect of empathy and consistently showed activation of somatosensory cortices, including anterior insula and anterior cingulate cortex, involved in experience sharing (Gazzola et al., 2012; Keysers et al., 2004; Rütgen et al., 2015, 2020; Wagner, Rütgen, & Lamm, 2020). In our study, the only cue to pain participants had was a short sentence describing a physically painful experience, which was unique for each participant. This task did not actively involve first‐hand pain; it instead engaged cognitive empathy mechanisms in building a representation of the target's mind.

### 4.1 Limitations

The pattern of correlations between the neural correlates and the empathy rates was not robust. Although this does not undermine the core results of our experiments, we could not establish whether empathy is directly drawn upon AM. It is important to further highlight that each participant was exposed to a total of only four contexts, one per experimental condition. This was done to optimize the training session of the classifier as we wanted to test the reactivation of specific memories when the target of empathy experienced that same event. However, this could limit the generalizability of the results to any kind of AM involvement in empathy. Furthermore, we have only tested Caucasian participants exposed to Caucasian stimuli and did not specifically test other factors shown in previous studies to be important modulators of empathic response (Jankowiak‐Siuda, Rymarczyk, Żurawski, Jednoróg, & Marchewka, 2015; Sessa & Meconi, 2015). Previous studies have repetitively shown cross‐ethnicity bias in neural empathic response (Avenanti et al., 2010; Sessa, Meconi, Castelli, & Dell'Acqua, 2014; Sheng & Han, 2012; Xu et al., 2009). These studies guided our experimental choice to focus on memory in the service of within‐ethnicity empathy. Although exploring cross‐ethnicity effects in how memory is involved in empathy would go beyond the intent of the current study, it is worth mentioning that this aspect could limit the generalizability of our results.

## 5 CONCLUSIONS

The present study provides important evidence of a re‐activation of AMs in the context of empathy. However, puzzling previous evidence showing little empathy impairment in patients with amnesia opens future research questions on whether memory causally drives empathy judgements. This would require future work that modulates memory retrieval in a time‐sensitive manner.

## CONFLICT OF INTEREST

The authors declare no conflict of interests.

## AUTHOR CONTRIBUTIONS

**Federica Meconi**: Formulated the research question, collected and analysed all the data, manually drew the ROIs around the hippocampus, and wrote the manuscript. **Federica Meconi**, **Simon Hanslmayr**, and **Ian Apperly**: Designed the studies. **Simon Hanslmayr** and **Ian Apperly**: Supervised the analysis and substantially contributed to the writing of the manuscript. **Catarina S. Ferreira**: Helped with the analysis of the fMRI data. **Bernhard Staresina**: Supervised the fMRI analysis and the drawing of the ROIs. **Juan Linde‐Domingo** and **Sebastian Michelmann**: Helped with the classifier and bootstrapping analysis. All the authors gave important feedback and comments to the manuscript.

## Supporting information

**AppendixS1:** Supplementary Information

## Data Availability

The study was not formally pre‐registered, but the data from this research are available to view in the OSF repository: https://osf.io/9z2uf/.
