# Supplementary material for: EEG and fMRI evidence for autobiographical memory reactivation in empathy
Source: Hum Brain Mapp. 2021 Jun 14;42(14):4448–64. doi: 10.1002/hbm.25557 (PMC8410563; doi:10.1002/hbm.25557)
Supplement: Supplementary file 1 — AppendixS1: Supplementary Information [file HBM-42-4448-s001.docx]

**Supplementary Materials**

1. **Methods**
   1. Questionnaires.

Participants also completed the Autobiographical Memory Questionnaire (AMQ; Rubin, Schrauf, & Greenberg, 2003) for their autobiographical episodes. The AMQ is a useful tool to collect ratings about the vividness of the memory, the accuracy with which participants can recollect and relieve the event and the date of the memory event.

With the aim of providing a validated score of the self-perceived empathy range of our sample of participants, the Empathy Quotient (EQ; Baron-Cohen & Wheelwright, 2004) was collected at the end of the experimental session. The EQ comprises 60 items including 20 filler items. Participants respond on a 4-points scale that ranges from “strongly agree” to “strongly disagree”, the score ranges from 0 to 80 (low: 0–32, middle: 33–52, high 53–63, extremely high: 64–79, and maximum empathy: 80).The Interpersonal Reactivity Index (IRI; Davis, 1983) was also administered to collect a finer-grained picture of the empathy traits, both affective and cognitive aspects. The IRI comprises 28 items subdivided in four subscales, two underpinning the affective component of empathy (i.e., the empathic concern, EC, and the personal distress, PD) and two underpinning the cognitive component of empathy (i.e., the perspective-taking, PT, and fantasy subscales, FS). Responses are given on a 1 to 5-points scale; no ranges of normal or abnormal empathy are identified.

Finally, the 20 items Toronto Alexithymia Scale (Bagby et al., 1994) was administered to participants with the aim of showing that our participants had on average normal ability of describing their own emotions. This was important as previous studies showed that alexithymia patients have also impaired others’ emotions processing. Responses were given on a 1-5 points scale and the maximum possible score is 80. Alexithymia is shown with scores above 61.

S1.2. Experiment 1: EEG

S1.2.1. The pain decision task

During the practice session of the pain decision task each participant was told the definition of empathy in the present context “Empathy is the ability to share the others’ emotions in a way that if you observe somebody being sad, you feel sad as well. You don’t feel sorry for that person because that would be compassion. In the same way, if you see somebody not expressing any particular emotion, you are not asked to say whether this person looks nice to you because that would be sympathy. Empathy is then the ability to mirror the others’ emotions.

S1.3. Experiment 2: fMRI

S1.3.1. The pain decision task

In the adjusted paradigm of the pain decision task each trial started with a fixation cross of a variable duration (2, 2.2, 2.4, 2.5, 2.6, 2.8, 3secs) followed by the sentence that was on screen for 3 secs. The fixation cross between the face and the sentence was jittered between 1.5 and 2.5 secs in steps of 100ms. Both the face and the following fixation cross were lengthened up to 1 sec and both the rating and the pain decision task were self-paced but if no response was given within 3 and 2 secs, respectively, the task would continue to the next trial. Therefore, for this experiment we only analyzed trials for which a response for the rate was given.

S1.3.2. fMRI analysis

S1.3.2.1. Region of interest

A Region of interest (ROI) for the hippocampus was manually drawn for each participant for a direct investigation of the activation of this brain area in both AM and non-AM conditions against baseline. Baseline was manually computed as the mean activity of the whole trial.

S1.3.2.1. Regressors of no interest.

*Analysis related to the onset of the context*. The regressors of no interest included the first and third fixation crosses, presentation of the face, onset of the rating and of the pain decision question, the button presses.

*Analysis related to the onset of the face*. The regressors of no interest included first and second fixation crosses, presentation of the sentence, the onset of the pain decision question, the 2 button presses.

1. **Results**

S2.1. Experiment 1: EEG.

S2.1.1. Correlational analysis

Correlations have been explored between neural responses and explicit measures of empathy and emotional traits. We calculated the differential score for the explicit judgments of empathy rates between the empathy judgements given for the faces depicted in the AM context minus those given in the faces depicted in the non-AM contexts.

We computed ERP effects time-locked to the onset of the faces for the processing of facial expression (i.e., painful minus neutral faces), for the processing of the preceding memories (AM minus non-AM) and for the sum of these differential scores. We averaged the ERPs in the 0.6 – 1s time-window in four preselected pools of electrodes covering frontal and centroparietal regions. We calculated in total 13 correlations so we would only accept correlations with p-values smaller than 0.0038. We found correlations between the differential scores of the empathy rating (AM minus nonAM) and the ERP effects for the processing of the memories, r = -.423, p = .025 (r = -.519, p = .0055 after removing an outlier that scored more than 2SD less than average) and with the sum of the ERP effects r = -.486, p = .0088. Although, these correlations suggest evidence of a relation between neural responses and explicit measures of empathy judgments in the time-window when the classifier showed significant effect, none of them held under conditions determined by the multiple comparisons correction.

We have computed one additional correlation to test whether the effect for painful events for which participants did or did not have an associated AM were directly correlated with the average differential scores of their empathy judgements in these conditions. Namely, we have computed differences between the ERP amplitudes on the time-window between 0.6 – 1 s. Then, we computed the difference between the participants’ average empathy rates for painful AM and painful non-AM. We observed a significant correlation between the pain effect observed on the ERPs and the differential empathy rates (r = .443, p = .01), meaning that the more the ERP difference between painful events for which participants did have an associated AM and those for which they did not, the greater the empathy judgements for painful AM. Also in this case, although significant, this correlation does not survive the multiple comparisons correction and therefore does not robustly supports a direct link between neural correlates and behavior. Figure S1 shows the scatterplot of differential ERP amplitudes for painful AM vs painful non-AM and differential empathy judgements in these conditions.


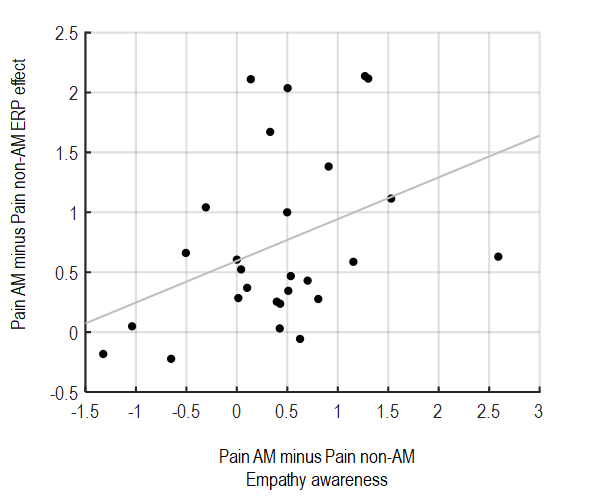


S1. Scatterplot showing the correlation between differential ERP amplitudes in 0.6 – 1s time-window for painful AM and painful non-AM and differential empathy judgements in these conditions.

S2.1.2. Behavioural results – Retrieval Task

Mean reaction times of vividness rates, mean proportions of vividness rates and of accuracy were inserted into a repeated measures ANOVA with emotion (painful vs. neutral) and memory type (AM vs. non-AM) as within-subjects factors.

For vividness rates, we observed a main effect of both emotion (*F*(1,27) = 12.235; *p* = .002, *η_p_^2^* = .312, *η^2^* = 0.05) and memory type (*F*(1,27) = 51.866; *p* < .0001, *η_p_^2^* = .658, *η^2^* = 0.528). On average, painful contexts were pictured with higher vividness than neutral memories (M_diff_ = .525, CI = [.217, .832]) and so was for the autobiographical contexts when compared with non-autobiographical contexts (M_diff_ = 1.711, CI = [1.22, 2.2]). The interaction was not significant (F < 1).

We observed a main effect of memory type (*F*(1,27) = 11.793; *p* = .002, *η_p_^2^* = .304, *η^2^* = .176) but not of emotion (*F*(1,27) = 1.273; *p* = .269, *η_p_^2^* = .045) on reaction times of vividness rates. On average, participants were faster in picturing an autobiographical than a non-autobiographical context (M_diff_ = .147, CI = [.059, .235]). The interaction between factors was also significant (*F*(1,27) = 6.165; *p* = .020, *η_p_^2^* = .186, *η^2^* = .029) such that painful autobiographical contexts drove faster vividness rates when compared to painful non-autobiographical contexts (*t*(27) = 3.89, *p* = .001, *d* = 0.74).

No main effect, nor interaction was observed for accuracy scores (max *F*(1,27) = 2.592, *p* = .119, *η_p_^2^* = .088). The accuracy was overall high and we report a table with means and standard deviations per condition.


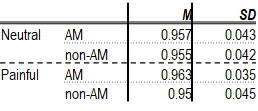


S2.1.3. ERPs – Retrieval task

Figure S2. ERPs time-locked to the onset of the cue and reflecting AM and non-AM (upper left panel), painful AM and painful non-AM (upper center panel) and neutral AM and neutral non-AM (upper right panel). In the bottom panels significant clusters observed in 1–2 sec time-window to the corresponding upper panels conditions. Colours code differential amplitudes between conditions.

S2.2. Experiment 2: fMRI

S2.2.1. ROI Analysis

The direct contrast between each condition and the baseline was performed in the ROI drawn around the hippocampus to account for its activation in both conditions. These tests showed that hippocampus was active in both AM (*t*(27) = 4.2492, *p* < .002) and in non-AM (*t*(27) = 4.7045, *p* < .00067) relative to a baseline that was computed as the mean run activation.

S2.2.2. Correlational analysis

Coorrelational analysis were performed to test for a link between behavioural and neural findings. We extracted activation in the 6 significant clusters of activation for the contrast AM > non-AM and non-AM > AM. We calculated individual differential empathy judgements for AM and non-AM (AM minus non-AM). Since 6 correlations were computed the accepted p-values was set to p = .0083.

Figure S3 shows the scatterplots for the significant correlations. A significant correlation was found between activation in the SFG and the differential empathy judgements r = .423, p = .025, does not hold after multiple comparisons correction. A significant and robust correlation was observed between the activation in the parahippocampal gyrus and the differential empathy judgements r = -.661, p = .0001.


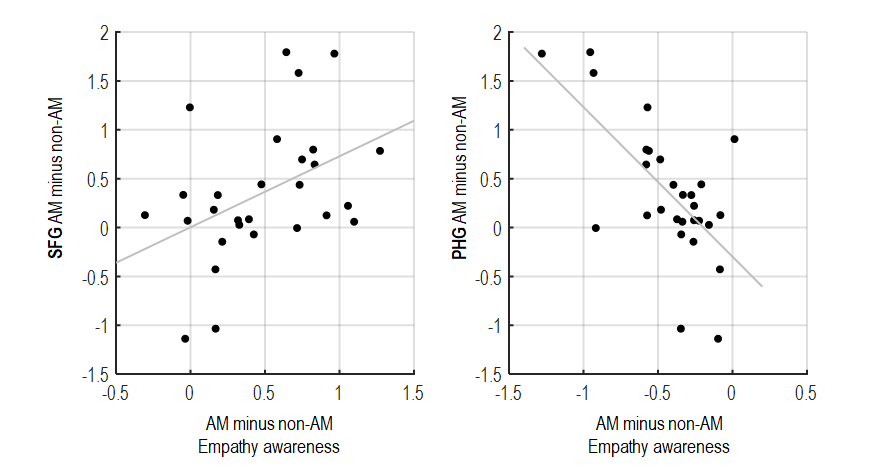


Figure S3. Scatterplots of the correlations between differential hemodynamic activation and individual differential empathy judgements. Differential scores for both hemodynamic activity and empathy judgements were computed as AM minus non-AM. Left panel: correlation between SFG activation and empathy judgements. Right panel: correlation between PHG and empathy judgements.

**References**

Bagby, R. M., Parker, J. D. A., & Taylor, G. J. (1994). The twenty-item Toronto Alexithymia scale—I. Item selection and cross-validation of the factor structure. *Journal of Psychosomatic Research*, *38*(1), 23–32. https://doi.org/10.1016/0022-3999(94)90005-1

Baron-Cohen, S., & Wheelwright, S. (2004). The Empathy Quotient: An Investigation of Adults with Asperger Syndrome or High Functioning Autism, and Normal Sex Differences. *Journal of Autism and Developmental Disorders*, *34*(2), 163–175. https://doi.org/10.1023/B:JADD.0000022607.19833.00

Davis, M. H. (1983). Measuring individual differences in empathy: Evidence for a multidimensional approach. *Journal of Personality and Social Psychology*, *44*(1), 113–126.

Molenberghs, P., Johnson, H., Henry, J. D., & Mattingley, J. B. (2016). Understanding the minds of others: A neuroimaging meta-analysis. *Neuroscience & Biobehavioral Reviews*, *65*, 276–291.

Rubin, D. C., Schrauf, R. W., & Greenberg, D. L. (2003). Belief and recollection of autobiographical memories. *Memory & Cognition*, *31*(6), 887–901. https://doi.org/10.3758/BF03196443
